# Picosecond to microsecond dynamics of X-ray irradiated materials at MHz pulse repetition rate

**DOI:** 10.1038/s41598-023-42943-z

**Published:** 2023-09-28

**Authors:** Vladimir Lipp, Jan Grünert, Jia Liu, Beata Ziaja

**Affiliations:** 1 Institute of Nuclear Physics, Polish Academy of Sciences, Radzikowskiego 152, 31-342 Kraków, Poland; 2 Center for Free-Electron Laser Science CFEL, Deutsches Elektronen-Synchrotron DESY, Notkestr. 85, 22607 Hamburg, Germany; 3 European XFEL, Holzkoppel 4, 22869 Schenefeld, Germany

**Keywords:** Techniques and instrumentation, Computational methods, Applied physics, Electronic properties and materials, Semiconductors, Nonlinear phenomena

## Abstract

Modern X-ray free-electron lasers (XFELs) produce intense femtosecond X-ray pulses able to cause significant damage to irradiated targets. Energetic photoelectrons created upon X-ray absorption, and Auger electrons emitted after relaxation of core-hole states trigger secondary electron cascades, which contribute to the increasing transient free electron density on femtosecond timescales. Further evolution may involve energy and particle diffusion, creation of point defects, and lattice heating. This long-timescale (up to a microsecond) X-ray-induced dynamics is discussed on the example of silicon in two-dimensional geometry. For modeling, we apply an extended Two-Temperature model with electron density dynamics, *n*TTM, which describes relaxation of an irradiated sample between two successive X-ray pulses, emitted from XFEL at MHz pulse repetition rate. It takes into account ambipolar carrier diffusion, electronic and atomic heat conduction, as well as electron-ion coupling. To solve the *n*TTM system of equations in two dimensions, we developed a dedicated finite-difference integration algorithm based on Alternating Direction Implicit method with an additional predictor-corrector scheme. We show first results obtained with the model and discuss its possible applications for XFEL optics, detectors, and for diagnostics tools. In particular, the model can estimate the timescale of material relaxation relevant for beam diagnostic applications during MHz operation of contemporary and future XFELs.

## Introduction

The latest developments of X-ray Free-Electron Lasers (XFELs)^1,2^ made it possible to generate ultrafast X-ray pulses sufficiently intense to strongly change the properties of target samples. This opened up a large number of new applications, such as diffract-and-destroy experiments^3,4^, particle imaging using Coulomb explosion^5^ or X-ray- induced photo-electrons^6^, material processing and nanostructuring^7^, catalysis^8^, biophysics^9^, and biomedicine^10^. Because of the complex multistage response of the material after the absorption of a femtosecond X-ray laser pulse^11,12^, computational models are essential to understand the material response, and for further advances in the field. One of the most important technical issues during X-ray irradiation experiments is to predict and eventually reduce possible damage to detectors, such as the high speed Adaptive Gain Integrating Pixel silicon Detector AGIPD^13^, which enables single pulse imaging for diffraction and scattering experiments at a 4.5 MHz frame rate. This requires a detailed knowledge of the X-ray-induced processes and dynamics.

One of the most advanced FELs, the European X-ray Free-Electron Laser, can provide up to 27,000 pulses per second at 4.5 MHz with the shortest delay between the pulses of $\sim$ 220 ns^14^ along with mJ-level pulse energy in the hard X-ray regime. Such a short inter-pulse delay raises questions of whether the X-ray detectors or the optical elements in the beamline can withstand the intense irradiation without a damage and relax after the excitation by a pulse before the arrival of the next pulse. Since future FELs like LCLS-II^15^ and SHINE^16^ are also expected to provide MHz pulse repetition rates, this issue may become even more crucial.

**Figure 1:**
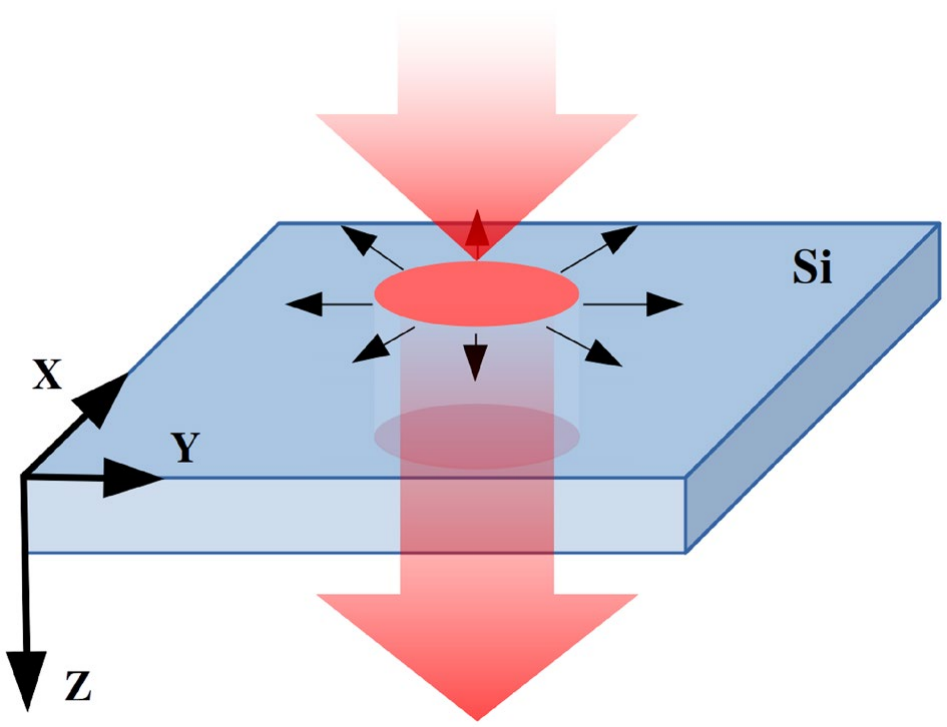
Normal incidence geometry in the X-ray irradiation experiments. Z is the direction of the incoming beam. X and Y represent coordinates in the plane lateral to the beam direction.

In a typical X-ray irradiation experiment, the beam is arriving at a certain incidence angle to the detector’s or optical element’s surface. Due to the attenuation of hard X rays in the material, the irradiated volume can be approximated as practically homogeneous in the direction parallel to the beam (in what follows called ’Z direction’) down to the photon penetration depth. I.e., the thickness of the material should be comparable or smaller than the photon penetration depth for this approximation to hold. Figure 1 shows the irradiation geometry in case of normal X-ray incidence, which we will analyze here. A generalization for the case of other incidence angles, including grazing incidence, is planned for future work.

In order to predict the dynamics of material cooling after an impact of an X-ray pulse with a certain parameter set, here we apply an extended Two-Temperature Model with electron density dynamics, *n*TTM^17^, which can describe the relaxation of a target on long timescales. It takes into account ambipolar carrier diffusion^18^, electronic and atomic heat conduction, as well as the electron-ion coupling. To solve the *n*TTM system of equations in two dimensions, we developed a dedicated finite-difference integration algorithm based on the Alternating Direction Implicit method^19^ with an additional predictor-corrector scheme. We show the first results obtained with the model and discuss its possible applications on preventing X-ray-induced damage of optics and detectors by pulses emitted from XFELs at high repetition rate. Moreover, the model can estimate the timescale of material relaxation relevant for photon beam diagnostic applications, e.g., in the so-called timing tool^20^ that provides information on shot-to-shot arrival time of X-ray pulses in respect to the optical laser pulses during the high-repetition-rate operation of XFELs.

## Model

The simulations presented in this work are based on the *n*TTM model, which was first introduced for the laser irradiation of silicon in Ref.^21^, then applied by one of the authors for the 1D case of ultrashort laser irradiation in Ref.^22^, and later described in detail (with the release of the corresponding software) in Ref.^17^. In this work, we extend the solver algorithm to two dimensions and apply it to predict energy diffusion in silicon after an X-ray FEL irradiation on up to 1000 nanosecond timescale. In what follows we will call the corresponding simulation tool ’NanoDiff’, as it is designed to simulate the diffusion of the energy and particle flows created by an X-ray pulse on the nanosecond timescales.

The model is based on the relaxation-time approximation of the Boltzmann equations^21,23^. It models the excited target as consisting of two subsystems: excited carriers (electrons and holes) and atoms, and follows their evolution. The atomic system is described with the atomic temperature $T_a$ obeying a simple diffusion equation, whereas the carrier system is modelled with two separate Fermi distribution functions that have the same temperature $T_e$ and density *n* but different chemical potentials, $\mu _e$ and $\mu _h$, respectively. Such an approach results in the following system of the nonlinear partial differential equations:

1 $$\begin{aligned}&\frac{\partial n}{\partial t}+\nabla \cdot \vec {J} = - \gamma n^3 + \delta \left( T_e \right) n , \end{aligned}$$

2 $$\begin{aligned} \quad C_{e-h} \frac{\partial T_e}{\partial t}&= - \nabla \cdot \vec {W} - \frac{C_{e-h}}{\tau _{e-p}} \left( T_e-T_a \right) \nonumber \\&\quad -\frac{\partial n}{\partial t} \left\{ E_g+\frac{3}{2}k_B T_e \left[ H_{\frac{1}{2}}^{\frac{3}{2}} \left( \eta _e \right) + H_{\frac{1}{2}}^{\frac{3}{2}} \left( \eta _h \right) \right] - n \left( \frac{\partial E_g}{\partial n} \frac{\partial n}{\partial t} + \frac{\partial E_g}{\partial T_a} \frac{\partial T_a}{\partial t} \right) \right\} \nonumber \\&\quad -\frac{3}{2} k_B T_e n \frac{\partial n}{\partial t} \left\{ \left[ 1- H_{\frac{1}{2}}^{\frac{3}{2}} \left( \eta _e \right) H_{\frac{1}{2}}^{-\frac{1}{2}} \left( \eta _e \right) \right] \frac{\partial \eta _e}{\partial n} + \left[ 1- H_{\frac{1}{2}}^{\frac{3}{2}} \left( \eta _h \right) H_{\frac{1}{2}}^{-\frac{1}{2}} \left( \eta _h \right) \right] \frac{\partial \eta _h}{\partial n} \right\} \end{aligned}$$

3 $$\begin{aligned}&\quad C_a\frac{\partial T_a}{\partial t}=\nabla \cdot \left( k_a\nabla T_a\right) + \frac{C_{e-h}}{\tau _{ep}}\left( T_e-T_a \right) . \end{aligned}$$

The notation used in Eqs. (1–3) is the following: $\vec {J}$ is the electron-hole (carrier) current, $\gamma$ is the Auger recombination coefficient, $\delta$ is the impact ionization coefficient, $C_{e-h}$ is the specific heat capacity of the excited electron-hole pairs, $\vec {W}$ is the ambipolar energy flow, $\tau _{e-p}$ is the electron-ion relaxation time, $E_g$ is the energy gap, $H_\zeta ^\xi \left( \eta _c \right) \equiv F_\xi \left( \eta _c \right) / F_\zeta \left( \eta _c \right)$ with $F_\xi \left( \eta _c \right)$ being the Fermi integral^17^, $\eta _{e,h}$ is the reduced chemical potential of the electrons and holes^17^, respectively, and $k_B$ is the Boltzmann constant. Eq. (1) describes the balance in the carrier number; Eq. (2) is the nonlinear diffusion equation for the carrier temperature and describes the energy balance in the excited free carrier system. The last equation, Eq. (3), describes the energy balance in the atomic subsystem. More details as well as the parameters used in the present simulations can be found in Ref.^17^.

In order to solve the above system of equations, we apply the modified Alternative Directions Implicit method^19^, which solves the equations in two substeps: first implicitly in X direction and explicitly in Y direction, and then *vice versa*. The method is coupled with the predictor-corrector algorithm, similarly as it was done in Ref.^17^. Recently, a similar approach was also applied to metals in two dimensions without accounting for the carrier density diffusion^24^. The equations are first linearized in $T_e$ by calculating all nonlinear terms at the previous time step. The predicted values are then applied in the next iterative step. In our model, the predictor-corrector procedure is repeated until the carrier temperature converges with the precision of $10^{-5}$ K.

Below we present the first results of the model and discuss their implications for the X-ray irradiation experiments.

## Results

As an application example of the NanoDiff code, we present the results of a simulation for a silicon bulk sample with the lateral size of 75 µm $\times$ 75 µm. The pixel size of the AGIPD detector at the European XFEL is 200 µm $\times$ 200 µm^13^, but we checked that the chosen simulated size provides identical results to a larger target while requiring a much lower computational cost. As mentioned above, we assume that the sample is homogeneous along the Z axis, which allows us to perform the simulation only in two dimensions. The silicon sample was irradiated with a hard X-ray FEL pulse arriving at normal incidence and focused at the geometrical center of the sample. The simulated beam focus was 10 µm (full width at half maximum), which is typical for detector irradiation at the modern XFEL facilities. For simplicity, we assumed that the initial electronic temperature and density at time $t=0$ followed a Gaussian shape in two dimensions with the full width at half maximum of 10 µm, the peak temperature of $T_e=42000$ K, and the peak carrier density of $n=2.5\times 10^{26}$ m$^{-3}$. These irradiation conditions correspond to the absorbed dose of 0.06 eV/atom at the pulse center, i. e., significantly below the structural damage threshold for silicon, which is about 0.65 eV/atom^25^.

The above initial conditions mimic the effect of an X-ray pulse with the photon energy of 4 keV (with photon penetration depth of about 9.6 µm^26^) and the fluence of 0.73 J/cm$^2$. They were obtained from an XCASCADE^27–29^ simulation, which gives the final electronic density at the end of the X-ray-triggered electron cascading^11^.

**Figure 2:**
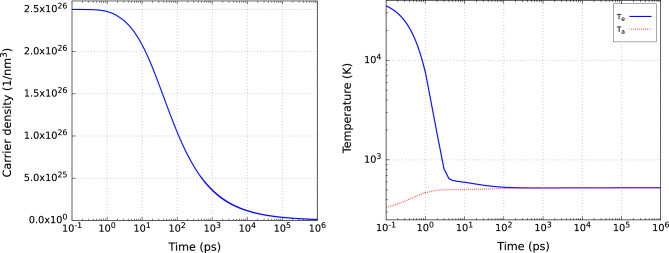
Time evolution of carrier density (left), and carrier and atomic temperatures (right) in silicon after an X-ray irradiation at the center of the focal spot. The initial electronic temperature and density were chosen to have a Gaussian shape in two dimensions with the full width at half maximum of 10 µm, the peak carrier temperature of $T_e=42000$ K, and the peak carrier density of $n=2.5\times 10^{26}$ m$^{-3}$, respectively. The time step was $2\times 10^{-14}$ s. The simulation box had the size of 75 µm $\times$ 75 µm with the grid size of 1.5 µm.

Figure 2 shows the behavior of the carrier density (left) and carrier/atomic temperature (right) up to the time of 1 µs, i.e., the typical delay between two consecutive X-ray pulses at the European XFEL in the 1 MHz regime. The plots show the evolution of the carrier density and temperature at the center as a function of time for the simulation timestep of $dt=2\times 10^{-14}$ s. We performed convergence studies of all results using smaller timesteps down to $10^{-18}$ s, see “Methods” below. They provided practically identical results to those obtained with $dt=2\times 10^{-14}$. Therefore, in what follows we use only the results obtained with the latter timestep.

**Figure 3:**
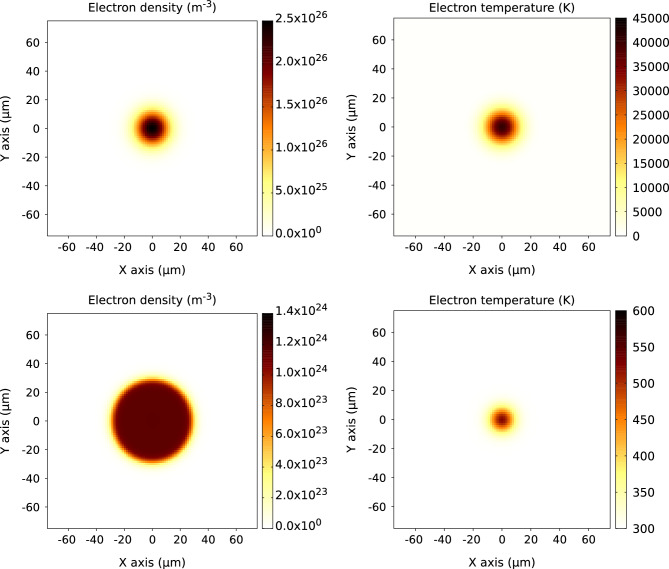
Carrier density, *n*, (left) and carrier temperature, $T_e$, (right) in silicon at $t=0$ µs (top) and at $t=1$ µs (bottom). The parameters of the simulation are the same as in Fig. 2. Note, that at $t=1$ µs, the electron and atomic temperatures are equal.

Figure 3 shows the corresponding contour plots for the carrier density and temperature at the beginning of the simulation (i.e., initial conditions) and at the end of the simulation. As we have already mentioned, the final distributions confirm that, under the chosen irradiation conditions, the sample would not be fully relaxed by the time when the next FEL pulse arrives.

## Discussion

The simulations show that the carrier density quickly decreases during the first nanosecond (Fig. 2 (left)). Afterwards, the decrease is slowed down, resulting in a much slower relaxation. Indeed, even by the end of the simulation at 1 µs, the sample did not relax to the ground state. This implies that the next pulse could drive the excitation slightly stronger, and a large number of consecutive pulses would lead to cumulative damage in the sample. This is confirmed by the second plot in the same figure (right) showing the evolution of the carrier (solid lines) and atomic (dashed lines) temperatures at the center of the focal spot.

As Fig. 2 (right) shows, the thermodynamic equilibrium between electrons and ions is quickly established, since the characteristic time of the electron-ion coupling used in the model is about 0.5 ps^17,30^. After that time, the electron and ion temperatures remain practically constant at the considered timescales. Although the carrier and energy diffusion, in general, should lead to the decrease of the temperatures in the focus center, the on-going Auger recombination also strongly affects the carrier dynamics, ultimately causing that the temperatures do not change there significantly on nanosecond timescales. This is caused by the large number of the excited carriers, which constantly recombine, increasing the carrier temperature. Consequently, even a slight increase in the temperatures occurs on the long timescales, according to the present model. The final atomic temperature is about 525 K, and the final carrier density is about $1.2\times 10^{24}$ m$^{-3}$.

The corresponding timescale on which the carrier density *n* changes is much longer, as other (much slower) processes determine this change. For the chosen simulation parameters (see Tab. 1 in Ref.^17^) and initial conditions, the Auger recombination (and not the carrier diffusion) plays the major role in the carrier density dynamics. Its rate is proportional to $n^3$ (Eq. 1, first term on the fight hand side), which eventually results in the homogeneous carrier density in the central region with elevated *n*, Fig. 3 (left, bottom). The carrier diffusion is proportional to the diffusion coefficient *D* (which is, in turn, proportional to the carrier temperature and has a very weak dependence on the carrier density, see Eq. (10) from Ref.^21^) and to the gradient of the carrier density (see Eq. (9) from Ref.^21^). As a result, the final carrier density distribution in the lateral plane, Fig. 3 (left, bottom), forms a circle, larger than the initial one, with almost homogeneous but low carrier density (two orders of magnitude smaller than the initial one).

Concerning the carrier temperature, Fig. 3 (right), the energy flux *W* plays the major role in its dynamics. *W* is proportional to the gradient of the carrier temperature (see Eq. (12) in Ref.^21^), which results in the relatively fast energy diffusion outside of the central region and, by time $t=1$ µs, smaller heated region than the initial one, Fig. 3 (right, bottom).

We are not aware of any available experimental results for a direct comparison with our model. Still, the timescales of the target relaxation obtained in our model look similar to the ones in the doped silicon irradiated with a femtosecond laser pulse^31^. More experimental data is required to confirm the model’s accuracy. The preparations for respective experiments are underway.

In the present example of the application of the NanoDiff code, we used the initial conditions estimated with our in-house simulation tool, XCASCADE. For more realistic initial conditions, in future, we plan to utilize our in-house code XCASCADE-3D^32^, taking into account ballistic electron transport^33^, beam polarization and a non-uniform spatial pulse shape. Its final energy and particle distributions can serve as the input for NanoDiff. Another planned improvement is to extend the code to three dimensions, which should allow to simulate grazing incident geometry and thus predict the relaxation of, e.g., optical elements at XFEL beamlines. It might also be useful for the femtosecond (optical) laser applications.

In summary, the NanoDiff code is a computationally efficient tool to simulate long-timescale relaxation (up to a microsecond) of carrier density, temperature, and atomic temperature in solid materials after impact of an XFEL pulse. It is a useful tool to analyze material relaxation at XFEL beamlines and detectors during MHz rate operation. The present simulations which show not fully relaxed silicon bulk after depositing a relatively low dose of 0.06 eV/atom in the center of the focal spot confirm the need for such analysis. We expect to report on the respective applications of the code soon.

## Methods

**Figure 4:**
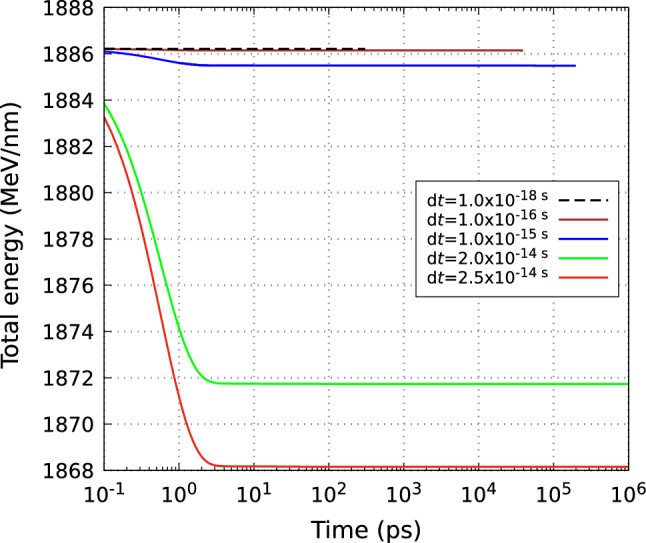
Evolution of the total energy calculated with different simulation time steps. Other simulation parameters are the same as in Fig. 2.

We performed the convergence studies confirming the reliability of the new simulation tool. Figure 4 shows the total energy as a function of time calculated for different simulation time steps. The calculations for time steps below $2\times 10^{-14}$ s did not reach $t=1$ µs due to high computational costs. As expected, for shorter time steps, the energy conservation is more accurate. The energy conservation scales linearly with the time step, as in the one-dimensional case^17^. Even for the longest time step considered, $2.5\times 10^{-14}$ s, the accuracy remains reasonable, Fig. 4. We have also checked that the evolutions of carrier density, temperature and atomic temperature practically do not depend on the time step within the time step range discussed above (not shown). For the final simulations, we used d$t=2\times 10^{-14}$ s. The full simulation up to *t* = 1 µs took about 11 days on a single core of Intel(R) Xeon(R) W-2225 CPU @ 4.10GHz, with the maximal number of corrections of 4 per direction and time step.

Similar convergence tests were performed for different grid and box sizes, confirming that the results obtained with the grid size of 1.5 µm and simulation box size of 75 µm $\times$ 75 µm (shown in the previous figures), are reliable. We also checked that setting the precision of the predictor-corrector to be $10^{-9}$ K instead of $10^{-5}$ K (which was used in the final simulations) leads to a negligible difference in the results.

## Data Availability

The simulation data that support the findings of this study are available from the corresponding author upon a reasonable request.

## Code availability

The NanoDiff code, which supports the conclusions within this paper and other findings of this study, will be available under a license agreement. The licensor is: Deutsches Elektronen-Synchrotron DESY, Notkestr. 85, 22607 Hamburg, Germany. Please contact the corresponding author for more details or check the following URL: https://xm.cfel.de/research/scientific_software.
